# “The system is well intentioned, but complicated and fallible” interviews with caregivers and decision makers about palliative care in Canada

**DOI:** 10.1186/s12904-021-00843-x

**Published:** 2021-09-22

**Authors:** Nicole Luymes, Nicole Williams, Liz Garrison, Donna Goodridge, Maria Silveira, Dawn M. Guthrie

**Affiliations:** 1 Department of Kinesiology & Physical Education, Wilfrid Laurier University, Waterloo, Ontario Canada; 2 Faculty of Social Work, Wilfrid Laurier University, Waterloo, Ontario Canada; 3 College of Medicine, University of Saskatchewan, Saskatoon, Saskatchewan Canada; 4 Division of Geriatric and Palliative Medicine, University of Michigan & GRECC, Ann Arbor Veterans Administration Medical Center, Ann Arbor, Michigan USA; 5 Department of Health Sciences, Wilfrid Laurier University, Waterloo, Ontario Canada

**Keywords:** Palliative care, Caregivers, Home care, Qualitative, Canada

## Abstract

**Background:**

Canadian palliative care (PC) philosophy seeks to support individuals in a person-centered and sensitive manner. Unfortunately, philosophy does not necessarily translate into practice and this divide may leave patients without appropriate care at the end of life, causing distress for some families. The primary goal of the study was to identify key factors affecting perceptions of quality PC from the perspective of informal caregivers and decision makers (e.g., program managers) and to understand how their experiences within the health care system may have influenced their perceptions.

**Methods:**

Nine caregivers and 11 decision makers from Yukon Territory, British Columbia, Alberta, Ontario, & Nova Scotia shared their experiences in PC via interview or focus group. Audio recordings were transcribed verbatim and qualitatively analyzed for themes.

**Results:**

Three themes emerged, including the Caregiver as Anchor, Bewildering System, and Patient, Caregiver, and Family-Centered Care. While these results resembled other studies on caregivers and individuals receiving PC, the present study also uncovered systemic concerns. There was agreement between the two participant groups across most subthemes, however only caregivers reported feelings of being trapped by the health care system and a general lack of respect from health care professionals. Additionally, caregivers stressed the importance of preserving some sort of normalcy in daily life despite the individual’s illness.

**Conclusions:**

Caregivers are critical. The health care system expects them to help a great deal, but they often do not feel supported or respected and the system is lacking the capacity and resources to meet their needs while they are grieving loss and struggling to meet demands.

## Background

Palliative care (PC) is an interdisciplinary approach to care that addresses the physical, psychosocial, cultural, and spiritual needs of individuals with a serious or life-limiting illness and their families [1]. PC supports individuals and their families through all stages of illness or decline and is not limited to just those who are imminently dying. PC engages all the relevant family stakeholders in decision-making to ensure the individual receiving PC receives care that is consistent with their values, beneficial (as opposed to burdensome), and feasible within the limitations presented by the individual’s body and his/her social support system [2].

Palliative care – whether through home-based palliative care or hospice teams – provides education to the caregiver, anticipatory guidance, material supports (e.g., supplies, oxygen, hospital beds), and respite care. Unfortunately, only 15% of Canadians have access to such services at home [3, 4]. Indeed, a recent population-level study of all decedents in Ontario found that PC services were infrequently delivered in a community setting, especially to individuals with life-limiting illnesses [5]. Although many benefits exist for community-based PC, few Canadians have actually received formal palliative care in their own homes [5–7].

In 2017, all provinces and territories in Canada made a commitment to a set of shared health priorities, which highlighted the need to improve access to home and community care, including palliative home care [8]. The need to improve access to PC provided in the home has also been acknowledged across a number of other countries, including Belgium [9], Italy, the Netherlands, Spain [10], the United Kingdom [11], and the United States [12]. Within Canada, the policies, services and delivery of home care is varied and depends on the needs of individuals as well as the resources available in each province. The level of care an individual receives is based on an assessment of their needs. While the delivery of PC services can differ across regions in Canada, there is a guiding framework which highlights that PC should be person- and family-centered, is integrated and holistic, with equitable access to care for anyone with a life-limiting illness [13]. Informal caregivers (i.e., unpaid caregivers who are typically family members or friends) are essential within this framework to allow individuals receiving PC to remain at home for as long as possible. Approximately 18% of Canadians have been informal caregivers to older adults, representing roughly $25 billion in terms of economic contribution [14]. Many caregivers have found satisfaction in this role [15–17] even though it has often been accompanied by stress and exhaustion [18, 19]. Caring for someone with a serious illness can be challenging as caregivers often make personal sacrifices (e.g., financial, employment, time) [20] to continue caregiving, which could lead to feelings of resentment and caregiver burden [21]. Caregiver burden has been associated with a number of negative health outcomes for caregivers, including worsening health, decreased quality of life, and increased mortality risk [22, 23]. It is therefore imperative for caregivers to have adequate supports allowing them to provide high quality care for individuals with a life-limiting illness.

The primary goal of this study was to identify key factors affecting perceptions of quality of PC from the perspective of informal caregivers and decision makers (e.g., program managers), as well as how their experiences within the Canadian health care system may have influenced these factors. While important steps have been taken to support caregivers in a person-centered and sensitive manner, this has not always translated into practice if health care systems and policy makers have competing priorities (e.g., financial considerations). Therefore, the second goal of this research was to compare the experiences of caregivers and decision makers to shed light on differences and similarities between these participants, so as to better understand how the health care system affects both groups. This study was part of a larger three-year project, funded by the Canadian Institutes of Health Research (CIHR), aimed at developing and testing quality indicators (QIs) for community-based PC. While the main goal of the larger project is to have a standard set of QIs for PC, as an initial step, we interviewed caregivers and decision makers about their experiences with PC to better understand what constitutes good quality care. The input from these individuals has assisted the research team in knowing what should be captured when developing QIs for PC.

## Methods

### Theoretical orientation

Because this study sought to understand the PC system from different points of view, the research was conducted and analyzed from a Systems Theory approach [24]. Systems theory examines patterns occurring at the system level (e.g., policy level decisions in health care) that directly and indirectly affect participants downstream, which – in the case of this research – include caregivers and individuals receiving PC.

### Participant recruitment

Knowledge users (e.g., an individual who uses research to make informed decisions about health policies, programs and/or practices) from five regions in Canada (Yukon Territory, British Columbia, Alberta, Ontario, and Nova Scotia) assisted with recruitment of individuals receiving PC and their informal/unpaid caregivers. Inclusion criteria for caregivers consisted of current or recent (within one year) care for an individual receiving palliative home care. Additionally, the knowledge users connected the team with decision makers within their health system’s PC leaders, including palliative program directors, managers, a team lead of home and community care and a provincial lead of palliative and end-of-life care practice. All decision makers had at least 2-5 years’ experience working in PC and the majority also had 5-10 years of experience working in home care. After several rounds of recruitment between January and October of 2019, nine caregivers, one patient, and 11 decision makers agreed to participate. Caregivers and decision makers represented all five provinces. The individual that was receiving PC was from Nova Scotia. While the original purpose was to describe the experience of those receiving PC and their caregivers, the research focused on the experience of caregivers due to the difficulty recruiting individuals receiving PC. To protect their privacy, all participants have been assigned pseudonyms.

### Data collection

Participants completed a background questionnaire to provide demographic information, followed by a semi-structured interview or focus group via telephone. Since caregivers were more likely to be speaking about their personal experiences with PC, while decision makers were speaking more broadly about working in PC, it was appropriate to conduct both types of interview styles based on the type of individual we were speaking with. We originally intended to complete all knowledge user communication using multiple focus groups, however due to time restraints and differences in time zones, it was difficult to schedule participants into focus groups. Therefore, some knowledge users ended up completing a one-on-one interview instead of participating in a focus group. All caregivers participated in a one-on-one interview, with the exception of one family in which both the caregiver and person receiving PC took part in the study.

### Data management

Interviews and focus groups were audio recorded, transcribed verbatim by LG and two research assistants, and supplemented with field notes (taken by the transcribers). To improve the accuracy of the transcription, transcripts were reviewed by each interviewee who could add, change, or omit data. While all received a copy of their transcript, only two caregivers provided new data in this process.

The authors analyzed all transcripts according to the methods of Braun and Clark [25], utilizing an inductive approach at the latent level. The coding guide was developed through an iterative process involving NL, NW, LG, DG, and DMG. NL, NW, and LG read each transcript several times and generated a list of codes. DG and DMG reviewed and further refined the codes based upon their reading of the data. NL, NW, and LG applied the coding scheme to all the transcripts using NVivo software. All transcripts were double coded at least once to ensure codes were used uniformly. The code book is available from the corresponding author (NW).

Code reports were generated and reviewed by NL, NW, and LG for emerging themes and subthemes. NL, NW, and LG also reviewed the themes and compared them against transcripts to ensure they were reflective of the dataset. NL sent a themes summary to all participants for feedback, to which seven caregivers and five decision makers responded in agreement of the findings. The remaining participants did not respond after multiple attempts at follow up. Triangulation between data sources (e.g., n=21 participants), methods (e.g., background questionnaire, interview, member reflections), and investigators (e.g., multiple researchers analyzed the data) [26] were also used to enhance reliability.

Of note, the coding guide was based upon interviews with individuals receiving PC and families and then applied to the data from decision makers. This was done for the following reasons: 1) the primary goal of this research was to understand the lived experiences of those receiving PC and their families, 2) caregivers and families were primary informants who reported on their lived experience, while decision makers were secondary informants; and 3) the patient and caregiver data were richer, allowing greater detail to illustrate emerging themes. As a result, the themes and subthemes were all saturated for the caregiver data (i.e., analysis continued until no new themes were identified), but saturation was not reached for some subthemes with regards to the decision makers.

## Results

### Subjects

Twenty-one individuals participated in this research: caregiver (n=9) and decision maker (n=11) subjects represented all five provinces; the individual receiving PC was from Nova Scotia. Of the nine caregivers, six cared for a spouse, one for a parent, one for a grandparent, and one for a friend; eight were female. Ten of the eleven decision makers were female; their positions included team/provincial lead (n=2), manager (n=5) and director (n=4; Table 1).

**Table 1:** Caregiver demographic data

M_age_ (range) – years	64.6 (25-81)
n female (%)	8 (88.9%)
M_length of time caregiving_ (range) – months^a^	50.6 (3-205)
Education	
Graduate Degree	3 (33.3%)
Undergrad Degree	3 (33.3%)
College Diploma	2 (22.2%)
High school	1 (11.1%)
Relationship to palliative patient	
Spouse	6 (66.7%)
Child	1 (11.1%)
Grandchild	1 (11.1%)
Friend	1 (11.1%)
Location of patient’s death	
Hospital – palliative	5 (55.6%)
Home	2 (22.2%)
Hospital – acute	1 (11.1%)
N/A	1 (11.1%)
Diagnosis of patient	
Cancer	3 (33.3%)
Kidney failure	1 (11.1%)
Heart failure and stroke	1 (11.1%)
Pneumonia	1 (11.1%)
Multiple system atrophy	1 (11.1%)
Heart failure	1 (11.1%)
ALS	1 (11.1%)
Medical Assistance in Dying	
Not mentioned in interview	5 (55.6%)
Utilized	2 (22.2%)
Plans to utilize	1 (11.1%)
Expressed interest, but did not receive	1 (11.1%)

^a^one participant provided care to multiple people, so time is unclear and not included in this value

Caregiver interviews lasted roughly one hour (range: 44 to 75 minutes). Seven decision makers participated in two tele-conference focus groups (Group 1 n=4; Group 2 n=3) and four decision makers were interviewed individually by phone to accommodate their schedules. Decision maker interviews and focus groups lasted approximately 40 minutes (range: 21 to 48 minutes). Three themes emerged upon analysis: Caregiver as Anchor, Bewildering System, and Patient, Caregiver, and Family Centered Care. Each theme was comprised of multiple subthemes, some of which differed between caregivers and decision makers (Table 2).

**Table 2:** Themes from caregiver data

Theme	Subthemes*
The Caregiver as Anchor	Part of the Team
	*Trapped by the System*
	*Lack of Respect*
	*Loss*
Bewildering System	Expectation vs. Reality
	Staffing
	Crisis
	*Exceptional Experiences*
Patient, Caregiver, & Family-Centered Care	Psychosocial and Spiritual Aspects
	Patient Choice
	Access to Care
	*Life Outside Diagnosis*

*italicized quotes were only saturated in caregivers, not in decision makers

### Theme 1: The caregiver as anchor

Caregivers were essential for ensuring patients received necessary services from the health care system. Without the caregiver as an anchor, the patient could be forgotten in the health care system and go without the supports necessary to maintain quality of life:

*As much as he [Carl, Sam’s husband] was having less of an anchor of life, the caregiver really then sort of then becomes the only anchor.* – Sam, caregiver

#### Subtheme 1: Part of the team

Caregivers listed tasks they completed on behalf of the patient, from activities of daily living (e.g., dressing) to medical support (e.g., administering medications). They were needed by patients, as well as PC teams, to act as a liaison between the two. Caregivers reported being relied upon by the PC team as the primary support for patients, and likewise, decision makers reported caregivers as valuable advocates for patients.

*I think that they [caregivers] should be looked at as truly part of the team.* – Alice, caregiver

Another described the important care coordination role of caregivers as:

*… just being on top of things, and recognizing that the system is fallible and, um, well, well-intentioned. It's a complicated system.* – Theo, caregiver

Decision makers recognized the reliance of PC on informal caregivers:

*… caregivers might be the best data source for a lot of those things [patient information], particularly when you get to end-of-life, because patients aren’t going to be talking for, speaking for themselves.* – Andy, decision maker

Caregivers felt a sense of duty to provide assistance to patients, but were also overwhelmed by the expectations of the health care team. Caregivers and decision makers acknowledged the importance of open communication to ensure adequate care for patients, while also supporting caregivers.

#### Subtheme 2: Lack of respect

In spite of acting as part of the health care team, caregivers recalled repeatedly explaining the patient’s needs to various health care professionals, which was draining and frustrating. Caregivers often felt a lack of control when interacting with the health care system and professionals. One caregiver described an episode with home care where they did not accommodate a change in schedule that would have benefited the patient:

*And it was, it was really, really, really difficult because they would insist. And there's times when they came to the door, “Well, if he’s not gonna go to bed now at 8:30, I'm not coming back” and they go and they’d leave me* – Adele, caregiver

Similarly, another caregiver described an instance when health care professionals seemed to disregard her husband’s wishes during his last days in the hospital, making her feel disrespected:

*…[the doctor] started telling me about how we wouldn’t keep a dog alive, when they’re going through suffering and pain. And I said, you know, “Don’t do this. I know what you’re saying. This is****his****[emphasis] decision. If it were mine, we would still be in hospice.” So, I had to, I won’t say fight, but I had to defend myself against the hospital, medical people.* – Norma, caregiver

Many caregivers described instances where they were not valued or respected. While Lack of Respect was not saturated for decision makers, caregiver recognition was discussed:

*I wonder if it would be important to measure just how often the caregivers are being engaged about or being asked the question ‘so how are you doing?’, uhm like I don’t think it’s happening as often as we would like to think it is.* – Taryn, decision maker

#### Subtheme 3: Trapped by the system

Similar to Lack of Respect, this theme emerged from caregivers only, as it specifically pertained to their feelings. Caregivers described feeling trapped in the home, hospital, or other physical space with the person to whom they were providing care. Caregivers were “lucky” to do necessary errands, let alone get a haircut or go for a coffee with friends given the precarious health status of patients.

*I couldn’t shop. If I ran across the street to the drug store it was running there and running back. Make sure that he’s gone to the toilet before I left. And, and hope that he’s still sitting in his chair when I get home and not flat on the floor*. – Joan, caregiver

As a result, caregivers’ worlds seemed smaller, with little to no social life and/or travel. Part of the concern was the inordinate amount of time spent waiting for appointments, medications, deliveries, etc., due to PC needs.

*We spent so much time in hospitals and doctor’s offices. If you looked at our calendar. That was our social calendar.* – Norma, caregiver

While the caregiving role was taken on willingly by the participants in this study, the role was described similar to imprisonment (a phrase selected by the authors): there was little room for caregiver’s autonomy outside of caregiving. Some caregivers appreciated the good they were doing, while others expressed resentment for lack of advance warning regarding the negative impact caregiving would have:

*I know it sounds lovely, and it sounds great. And whatever. But I thought to myself. No one told you, me at least, or maybe society, the burden is basically going to be on****you****[emphasis] to deal with****everything****[emphasis].* – Sam, caregiver

Like Sam, other caregivers noted disillusionment in their role, not necessarily because they did not wish to be a caregiver, but because they did not realize the extent of isolation and responsibility in PC. Caregivers wished they were informed of the burden they would carry alone, and the impacts caregiving would have on their freedom, social lives, and work. The Lack of Respect and Trapped by the System subthemes both highlight the importance of providing patient- and family/caregiver-centered care, which is essential when providing high-quality PC.

#### Subtheme 4: Loss

Caregivers provided care while navigating the loss of their future life with the patient:

*I’m also grieving my husband. I’ve been grieving him, like I tell the children, a long time, because I’m losing him, I’ve lost him slowly, right?* – Helen, caregiver

Some caregivers utilized professional supports to traverse their complex grief and bereavement, while others did not deem it necessary due to other support systems (e.g., religious communities). Decision makers recognized the emotional and psychological burden of grief and loss on caregivers, as well as shortfalls within the health care system to support these feelings:

*Often what I find is falling short a bit is the awareness piece around how the family felt, even in the bereavement period. You know, is the care ongoing? Do they still feel supported? Do they have the bereavement support that they need? And how was their experience of this end-of-life care and through death overall?* – Nancy, decision maker

Interestingly, decision makers commented on providing caregiver and/or family support *after* the patient had died (Nancy’s quote above), while caregivers expressed desire for support *during* PC:

*You're immersed in a grieving process ‘cause you're already grieving the loss of this loved one. If you were a visitor to a patient, a palliative patient, you're already, probably, in these complicated emotions and are feeling a whole range of thoughts in grieving. You're grieving already.* – Theo, caregiver

Caregivers described grief as anticipatory, while decision makers discussed grief as something to address after the patient had died. This theme clearly highlighted the need for additional bereavement care earlier in PC, especially for primary caregivers, who must balance full-time care with anticipatory grief.

### Theme 2: Bewildering System/Navigating the System

The health care system was described as complicated by caregivers and decision makers alike. Sam described the expectation for him to make medical decisions when experiencing exhaustion as a result of his caregiving:

*How would we have entered into [decision making] if your caregiver actually is sufficiently exhausted enough that you actually question my decision making? I found that very bewildering. I found that very, um, confusing*. – Sam, caregiver

There were many instances where PC did not meet patient or caregiver needs, which caregivers described as distressing. Caregivers were unsure how to traverse the system, which was neither intuitive nor user-friendly.

#### Subtheme 1: Expectation vs. Reality

This subtheme identified a lack of congruence between caregiver expectations and available service. The caregiver (and patient) expected a certain amount of support from health care providers; however, not all of these expectations were realized. While communication would have identified discrepancies, caregivers reported few discussions with health care professionals regarding PC:

*I do think that it’s really important sitting down at the beginning and just****talking****[emphasis] about, what they can do, what my expectations were, can the, is it realistic, if not, what can we do, what’s out there, what’s available, you know?* – Alice, caregiver

Decision makers wanted to support patients and caregivers, but recognized limitations within the health care system. A decision maker described her experiences with caregiver expectations in home care:

*I can recall this conversation actually with a daughter when I was in rural [care], surprised when you know we said we can only do this much and she’s like “you’re expecting me to take care of my mother?” … people don’t understand home care and that it’s a support to the family, in uhm supporting them to take care of their family member. … But the reality of what that actually looks like, people are completely unprepared for that*. – Beth, decision maker

Unfortunately, caregivers had negative experiences within the system and with health care professionals, such as sub-optimal patient placement. For example, Theo felt it was inappropriate for his aunt to die in a shared hospital room (opposed to private):

*And I felt sorry for the three other ladies who had no mobility, couldn’t run because they were, you know, bed bound often... I mean [my aunt] was, she was allowed to die there. I thought what, this is****awful****[emphasis]. I mean whoever made these decisions, that it was convenient for them not to have to move around or find a bed. That was really, really inappropriate.* – Theo, caregiver

While person-centered PC was a goal identified by decision makers, caregivers provided numerous examples where the health care system fell short of their expectations (e.g., Theo’s quote above). This problem was due to incongruence between caregivers’ expectations and the reality of PC in the Canadian health care system, as well as lack of dialogue around said realities.

#### Subtheme 2: Staffing

Various concerns existed in PC due to the limited number of professionals and services available, which were identified by caregivers and decision makers alike. Decision makers expressed concerns with limited resources, specifically staff:

*And the other piece is just the resources are not available, whether that is funding, or competent, um, personnel. For example, do you have a physician who knows how to deliver these services on a regular basis? Not necessarily a specialist. Are they even in the area? So, that’s a multi-factorial, kind of thing.* – Andy, decision maker

Caregivers (and patients) expressed lack of continuity between and support from health care professionals as challenging:

*There just was no continuity. So it was, um, uh. That, that was certainly the biggest challenge. It affected, it affected, like I said, all, all of the care he received.* – Alice, caregiver

Staff competency was addressed by participants as negatively affecting caregiver and patient experiences at the end-of-life.

#### Subtheme 3: Crisis

Crisis situations arose when patients required an ambulance and/or emergency department to get needed care. This theme applied to both caregivers and decision makers, but the experiences of each group differed. Crisis was distressing and overwhelming for caregivers, who turned to emergency services when there were no alternatives:

*Then there were all the calls for ambulances to get him to the hospital. I tried getting him there myself by taxi, at first. But eventually I gave up and started calling ambulances*. – Joan, caregiver

In some cases, caregivers felt crises were the only means for conveying patient needs to health care professionals. Conversely, caregivers may not have explored other options, which could have preceded or resulted from exhaustion:

*I know that there is respite available, but it wasn’t offered, and I just didn’t think to ask. I think, to be honest I was really in crisis and I was just getting through the day*. – Alice, caregiver

It was common for patients to experience crises that required immediate attention; however, many crises could have been avoided:

*I think some, you know, introductory understandings to palliative care. You know, that idea of advanced care planning, serious illness conversations, uhm because I think if you do that work up front, you’re gonna probably end up in less crisis.* – Beth, decision maker

With increased communication and care planning, decision makers felt crises could be reduced.

#### Subtheme 4: Exceptional experiences

Despite many concerns with PC, caregivers reported some positive experiences. This subtheme did not apply to decision makers. These exceptionalities typically pertained to specific circumstances, such as receiving PC at home and hospice.

*And I'll be honest with you, [our palliative care coordinator] has been tremendous advocate for me. She really has.* – Adele, caregiver

The PC coordinator ensured the couple’s needs for home care were met. Similarly, one participant described a home care aide who supported her husband, in turn giving her more freedom:

*And oh, the care aide. What really helped our life a lot too, was in the last 2 years we got a care aide and same care aide to come every time. And he was like an****angel****! [emphasis] He, he got to know Henry really well. Henry trusted him. Henry didn’t mind me leaving when he was with him.* – Norma, caregiver

It was especially important for this individual to have the same aide over time to establish a trusting relationship. Positive occurrences were out of the ordinary and did not outweigh distress from understaffing, lack of continuity, or other stressors associated with PC:

*…once she was in [the long-term care facility], it was a good experience. But it was just getting to that point and everything.* – Robin, caregiver

Exceptional care was relatively uncommon in caregivers’ PC experiences, partially due to health care system concerns, in addition to caregiver burden and complex grief.

### Theme 3: Patient, Caregiver, & Family-Centered Care

Decision makers and caregivers stressed the importance not only for patients, but also caregivers and family members to have their wishes and needs respected in PC. A caregiver described how this affected her experience:

*… he was put into hospice because there you’d, they keep him comfortable. And I have to tell you it was like heaven for me. Because I got to be his wife, not his caregiver. And I spent a lot of time talking with him.* – Norma, caregiver

Unfortunately, many did not experience patient, caregiver, and family centered care.

#### Subtheme 1: Psychosocial and spiritual aspects

Participants reported psychological, social, and spiritual aspects affected quality of PC. Caregivers experienced psychosocial distress, such as intense anxiety, while decision makers recognized significant effects associated with death and dying. One participant discussed the psychological aspects of his diagnosis:

*I went through what probably a lot of people do, “why me?” Because I lived a pretty healthy lifestyle and so on, and I couldn't figure out why this was happening to me. Well, I guess if I could figure it out, we might have a cure for what’s going on. But, uh, it’s been tough. Like anything I had my ups and downs, and that. But I’ve gotten by the “why me” stage and that now, you know I accepted what’s happening and to be honest we do it one day at a time, how we feel.* – Will, palliative patient

Accepting a life limiting diagnoses was challenging, particularly when needs were not being met:

*… he definitely had, um, um, emotional and psychological needs that weren’t being met. I think he felt very alone. And I know there were times I felt very alone*. – Alice, caregiver

Psychosocial and spiritual needs were unaddressed in the health care system, not necessarily due to lack of consideration, but rather due to competing priorities, such as patient pain.

*we always, you know uhm, pay a lot of attention, or give a lot of attention to pain and symptom management, but the bereavement part, or the spiritual part …We talk about it and it’s in our standards and all that sort of thing, but we really don’t put a huge emphasis on it. Like as much as it deserves.* – Carrie, decision maker

In some cases, spiritual and religious practices mitigated psychological and emotional distress:

*I had friends try to come, in case I needed to talk and stuff… I had everything I needed in my religion and in my time with Henry in the hospice. If I’d been in the hospital the whole time, I might of needed grief counseling afterwards.* – Norma, caregiver

However, there were circumstances where patients wished for spiritual care in a hospital or home care setting, but they were unable to receive such supports. Of note, few caregivers discussed their spiritual needs beyond the provision of specific religious guidance or rituals.

#### Subtheme 2: Patient choice

Participants stressed the importance for patients to express their wishes and have said wishes respected in PC. Toward the end of life, some patients lost their ability to make decisions and/or communicate their needs. Respecting a patient’s wishes was described as imperative:

*… the only thing I would compare it to you know you’re giving birth, you’re in control. It's your birth, birthing. You call the shots. And I think it's your death, you call the shots*. – Theo, caregiver

Decision makers also highlighted the importance for patients to specify their preferred place of death, who provides their care, where care is received (e.g., hospice), what type of care is delivered, etc. to their caregivers while they still have the cognitive capacity to do so:

*…we want to make sure we have a documented signed uh, substitute decision maker form. Uh, because often times you get into trouble if you’re not talking to the right person or once the person has lost capacity then things get a lot more difficult.* – Carrie, decision maker

However, caregivers reported instances where patient wishes were not respected, even when clearly communicated to health care professionals. For example, one individual was questioned for his decision to forgo life-extending procedures:

*And I think that you could feel that you know, Carl was walking down a different path, and sometimes they [medical professionals] got frustrated with him, that he wouldn’t participate in a medical solution.* – Sam, caregiver

Participants felt medical assistance in dying (MAID) provided patients with some control over their lives, rather than lifesaving or life-extending interventions.

*… just knowing myself and just my personality and so on. I just don't want to go the route where I struggle for my breath each day, you know, and that type of thing. So, I don't want to say it’s the lesser of the two evils. I just feel that it’s best for me. That’s the way I want to go.* – Will, palliative patient

Patient choice regarding medical intervention, place of death, and final wishes was clearly expressed by caregivers and decision makers as important in quality PC.

#### Subtheme 3: Access to care

Caregivers listed PC, access to resources, and appropriate care facilities as important for positive palliative experiences; however, there were difficulties in accessing PC:

*… we even had to really advocate, push very hard to get a palliative physician on board. Um, um [pause]. And so certainly once we got a palliative physician, the pain management was better.* – Alice, caregiver

This couple knew which type of care they wanted and worked to gain access to resources (e.g., transportation, home care); however, patients and caregivers may not be aware of services available:

*You know, are they not accessing it because it’s not available, or are they not accessing it’s because it’s not needed, they’re not made aware of the services, or the decision to not move to palliative care is not made in a timely manner? You know, there’s various reasons behind these access questions.* – Elise, decision maker

While many services were available to PC patients and caregivers in some areas, others were unavailable, especially in rural and remote regions in Canada. Caregivers reported lack of awareness of PC services, which reduced their access to care.

#### Subtheme 4: Life Outside diagnosis

Caregivers and patients experienced life changes due to diagnoses and health decline; however, they wanted their lives to continue as normally as possible for as long as possible. This subtheme emerged only from caregiver data.

*I think for a lot of people trying to live as normally as they can for as long as they can is, is, uh really important.* – Alice, caregiver

Caregivers and patients sought rounded treatment: they wanted high quality of life for as long as possible, but there were barriers in the extent to which they could live “normally”, in part due to instances of impersonal care. Adele and Will noticed a diagnosis-centric focus in their home care providers, who offered little support for their day-to-day activities and expected them to be home, waiting for care. For instance, Adele disliked when health care providers referred to her husband as his diagnosis:

*he might have ALS but he's still an individual, he’s still a person we're still a family. Husband and wife* – Adele, caregiver

It was important for caregivers and patients to be able to live with dignity, especially in the time leading to more intensive PC.

## Discussion

Three themes emerged upon analysis of qualitative data from caregivers and decision makers in Canada, including the Caregiver as Anchor, Bewildering System, and Patient, Caregiver, and Family-Centered Care. While these results resembled other studies on caregivers and individuals receiving PC, both in home and hospital settings [27–29], the present study also uncovered systemic concerns. There was agreement between the two participant groups across most subthemes, however only caregivers reported feelings of being trapped by the health care system and a general lack of respect from health care professionals. Since decision makers are not directly accessing the health care system, it is not surprising that these two subthemes were not saturated for this group. However, these two subthemes do highlight the importance of how caregivers had a poor experience with the health care system and, as a result, their perceptions of the quality of care that was received was sub-optimal. Additionally, caregivers stressed the importance of preserving some sort of normalcy in their everyday lives despite the patients’ illness.

Much research has uncovered the extent of stress and exhaustion accompanying the caregiving role, particularly in PC [30], and thus the focus of this research was to examine systemic patterns that may have influenced caregiver experiences. An element that may have affected caregiver distress was the stark difference between caregivers’ expectation of PC and the reality of the health care system as reported by decision makers, which functioned within logistical bounds, such as physical and financial resources. Although both caregivers and decision makers discussed similar aspects of what constitutes good quality PC, the lens on how this care is provided was vastly different between these two groups. For example, decision makers would often describe the “ideal” way to provide PC, such as having open and continual communication with the family, providing access to supports and services and bereavement care. Conversely, the actual experience that caregivers described did not match this “ideal” scenario. Most caregivers felt like the did not have the knowledge/information they needed to provide care and oftentimes had to advocate on behalf of their loved ones. Therefore, there was a clear disconnect between what the system could provide and what caregivers expected from the system.

Previous studies reported that caregivers felt expectations to “be in charge” of the patient to a greater extent than anticipated, causing distress [31]. This is especially true as the individual gets closer to death as a recent study found that the intensity of care provided by informal caregivers increased as the individual approached death. Care needs of individuals are they approach death are likely to increase and in turn, the number of hours of care provided by informal caregivers will likely also increase [32]. Caregivers in the current study similarly felt it was their duty to be part of the health care team, but also experienced a lack of respect from health care professionals, as well as feeling trapped in their role. There are numerous ways in which the health care system can support caregivers in their role including providing access to available services (e.g., respite, bereavement), education/training on how to care for someone at home and providing open and continual communication throughout the illness trajectory to ensure everyone’s needs and expectations are met [33, 34].

Most Canadians have indicated they would prefer to receive care in the comfort of their own homes and also to die at home, surrounded by loved ones [35, 36]. However, providing care at home is associated with various challenges for caregivers, including administering medications, providing transportation to appointments, and assisting with activities of daily living. Most caregivers lack formal training in caring for individuals with serious illnesses, increasing the likelihood for caregivers burden [37]. Many caregivers in the current study expressed frustration with the health care system as they did not feel supported in their role and lacked important information on how to care for the patients. A recent scoping review noted similar health system issues as caregivers did not have the basic information they needed to provide care at home, or the knowledge of how to access resources and services that could assist them in this role [38]. Since PC should be both patient- and family-centered, health care teams must provide adequate support and anticipatory guidance to caregivers, ensuring they feel prepared in their role and have access to necessary resources.

The caregivers in this study experienced difficulties with accessing timely PC, and oftentimes had to advocate on the patient’s behalf. Until recently, most PC services have not been initiated until the last weeks or days of life; however, the PC community has shifted towards providing PC services earlier to anyone who could benefit from this approach to care. This shift has been observed in multiple countries, including Canada, Australia, the United Kingdom, the United States, Belgium and New Zealand [9, 11, 12, 39–41]. Access to services can start early in the illness trajectory and occur concurrently with life-prolonging treatments. The initiation of PC services earlier in the illness trajectory has the potential to reduce health care service use (e.g., hospitalizations), reduce symptom burden, and improve overall quality of life for both individuals and families [42, 43]. A palliative approach to care supports the notion that both patients and families should be supported throughout the entire spectrum of care, including continual and on-going discussions about their wishes, goals, and care needs with the health care team. While the health care system in Canada has moved towards this approach, obstacles remain (e.g., resource availability, lack of hospice beds), as noted by decision makers.

### Study strengths and limitations

There were a number of limitations with the current study. First, the small sample may not be representative of patients, caregivers or decision makers nationally. There were many impediments to recruitment of patients including mental status and limited life expectancy. However, research has found that caregivers have provided adequate proxy reports of patient symptoms and areas of distress (e.g., insomnia, pain, etc.) [44], and had moderate agreement with patients around overall quality of life [45]. While there was more success recruiting caregivers and decision makers, diversity was limited: most participants were white, educated, and English-speaking.

There were also a number of strengths in this study, including timely recruitment of caregivers (e.g., current caregiver status, or within one year of patient death) in order to avoid gaps in memory. Additionally, the inclusion of both family caregivers and decision makers provided a perspective that has rarely been explored. While a number of studies have examined patient and caregiver experiences [31, 33, 46] as well as care providers and other stakeholder experiences [47] in Canada, to the authors’ knowledge, no qualitative studies have compared the experiences of multiple stakeholders within a Canadian PC context. Lastly, rigorous qualitative methodology including a series of triangulation approaches were employed to ensure data credibility [26].

## Conclusions

Discussions with caregivers and decision makers about their experiences with PC in Canada was an important first step in understanding what constitutes good quality PC. Based on their experiences, it is clear that a number of factors need to be included in the quality assessment of PC provided: anticipatory guidance about caregiver’s role, delineation of responsibilities between system and caregiver, extent of collaboration with caregiver, patient-centeredness of care, treatment of patient as a whole person, continuity of providers, and quality and frequency of provider communication. These factors should be addressed at the systemic level given how pervasive the critique of the system was by caregivers. Frameworks and action plans towards better supporting informal caregivers in the home, including education, training and increased access to PC services are part of the top priorities for delivery of PC in Canada [48]. Continual and timely communication between patients, caregivers, and health care providers about the patient’s wishes, goals, and overall care needs is essential to ensure that both patients and caregivers feel supported and have the necessary information available to provide care in the home. While many services and supports are available, improved efforts for communicating these services (and what is realistic to expect) to patients and caregivers is vital to support caregivers and enable them to continue their important role.

## Data Availability

The code book used for analyzing the data in the current study is available from the corresponding author on reasonable request.
